# Fruit Quality Characterization of New Sweet Cherry Cultivars as a Good Source of Bioactive Phenolic Compounds with Antioxidant and Neuroprotective Potential

**DOI:** 10.3390/antiox9080677

**Published:** 2020-07-28

**Authors:** Fabiana Antognoni, Giulia Potente, Roberto Mandrioli, Cristina Angeloni, Michela Freschi, Marco Malaguti, Silvana Hrelia, Stefano Lugli, Fabio Gennari, Enrico Muzzi, Stefano Tartarini

**Affiliations:** 1 Department for Life Quality Studies, Alma Mater Studiorum—University of Bologna, Corso d’Augusto 237, 47921 Rimini, Italy; fabiana.antognoni@unibo.it (F.A.); giulia.potente@unibo.it (G.P.); michela.freschi2@unibo.it (M.F.); marco.malaguti@unibo.it (M.M.); silvana.hrelia@unibo.it (S.H.); 2 School of Pharmacy, University of Camerino, Via Madonna delle Carceri 9, 62032 Camerino (MC), Italy; cristina.angeloni@unicam.it; 3 Department of Agricultural and Food Sciences, Alma Mater Studiorum—University of Bologna, Viale Fanin 46, 40127 Bologna, Italy; stefano.lugli61@unimore.it (S.L.); fabio.gennari3@unibo.it (F.G.); enrico.muzzi@unibo.it (E.M.); 4 Department of Life Sciences, University of Modena and Reggio Emilia, Biology Building, Via Giuseppe Campi 213/D, 41125 Modena, Italy; stefano.tartarini@unibo.it

**Keywords:** anthocyanins, antioxidants, brain-derived neurotrophic factor (BDNF), neuroprotection, new sweet cherry cultivars, nutraceuticals, oxidative stress, *Prunus avium* L., SH-SY5Y cells

## Abstract

Sweet cherries (*Prunus avium* L.) are highly appreciated fruits for their taste, color, nutritional value, and beneficial health effects. In this work, seven new cultivars of sweet cherry were investigated for their main quality traits and nutraceutical value. The phytochemical profile of three classes of phenolic compounds and the antioxidant activity of the new cultivars were investigated through high-performance liquid chromatography with diode array detection (HPLC-DAD) and spectrophotometric assays, respectively, and compared with those of commonly commercialized cultivars. Cyanidine-3-*O*-rutinoside was the main anthocyanin in all genotypes, and its levels in some new cultivars were about three-fold higher than in commercial ones. The ORAC-assayed antioxidant capacity was positively correlated with the total anthocyanin index. The nutraceutical value of the new cultivars was investigated in terms of antioxidant/neuroprotective capacity in neuron-like SH-SY5Y cells. Results demonstrated that the new cultivars were more effective in counteracting oxidative stress and were also able to upregulate brain-derived neurotrophic factor (BDNF), a pro-survival neurotrophin, suggesting their potential pleiotropic role in counteracting neurodegenerations.

## 1. Introduction

Dietary habits play a crucial role in the prevention of chronic diseases such as cardiovascular diseases, cancer, diabetes, and neurodegenerative disorders [1]. Fruit and vegetables are essential components of the human diet, and their intake is associated with the maintenance of health and the prevention of a wide range of diseases [2].

Sweet cherry (*Prunus avium* L.) fruits are among the most widely consumed fruits in temperate areas. The annual global production is about 2.2 million tons, the market being led by Turkey, the USA, and Iran [3]. In 2017, Italy was the sixth biggest producer worldwide and the biggest in Europe; in Italy, Emilia-Romagna is one of the five most important production regions [3].

Despite the wide array of cultivars present in Italy, there is considerable interest in obtaining new ones that would extend the harvesting season. Among the most desirable fruit quality traits, there are very early and/or late ripening time, large cherry size, light or dark red color, firmness, sweetness, and taste [4]. Thus, several breeding programs have been conceived with the aim of releasing on the market new cultivars, possessing these kinds of improved quality attributes. For this purpose, more than thirty years ago the University of Bologna launched a sweet cherry breeding program, by crossing quality-selected European genotypes with self-fertile American counterparts [5], which allowed the release of the seven new cultivars here studied and characterized.

Sweet cherries are rich in simple sugars and analogues (glucose, fructose, and sorbitol), organic acids (malic and succinic acids), dietary fibers, hydrosoluble (B and C), and liposoluble (A, E, and K) vitamins [6]. In addition to the well-known protective role of fibers and vitamins, epidemiological studies have pointed out a strong relationship between plant secondary metabolites and human health [7]. Phenols, contributing to color, flavor, bitterness, and astringency of plants [8], constitute a large group of specialized plant metabolites and have attracted particular attention due to their health-promoting properties [9]. In sweet cherries, they consist mainly of anthocyanins that confer the characteristic attractive color to the fruit, hydroxycinnamic acid derivatives, flavonols, and flavan-3-ols [10]. All these metabolites are among the most important antioxidant compounds in cherries [11,12], and changes in their composition and concentration can be modulated by both genetic and environmental factors [13]. A lot of evidence highlights the important role of this endogenous antioxidant machinery in relation to fruit quality and shelf-life [14]. Producing cherry fruits rich in endogenous antioxidant metabolites could delay fruit senescence and preserve their nutritional/nutraceutical value during the shelf-life time span.

It has been widely recognized that phenolic compounds exert a protective effect against oxidative stress [15], and a growing body of preclinical and clinical research has identified various beneficial health effects connected to their intake [16,17,18]. Oxidative stress is an imbalance between reactive oxygen species (ROS) production and their degradation by the endogenous antioxidant system [19]. ROS accumulation leads to damages to essential biological macromolecules with deleterious consequences [20]. Oxidative stress is considered a common mechanism involved in the onset and progression of the most widespread chronic and degenerative diseases, such as neurodegenerative disorders. The brain is particularly exposed to oxidative stress due to its elevated oxygen consumption, high content of polyunsaturated fatty acids, and low levels of antioxidants [21]. Strong evidence suggests that oxidative stress plays a key role in important neurodegenerative syndromes, such as Parkinson’s [22] and Alzheimer’s diseases [23]. Neurotrophic factors, such as the brain-derived neurotrophic factor (BDNF), have also been extensively investigated in the context of neurodegeneration [24]. Patients suffering from Parkinson’s or Alzheimer’s diseases show lower levels of BDNF [25], and this is associated to an increase in degeneration of dopaminergic neurons in the former [26] and to memory impairment in the latter [27]. In this context, the modulation of BDNF has emerged as another promising therapeutic approach to counteract neurodegeneration, and anthocyanins have been suggested as potential modulating agents [28].

The aim of the present work is to characterize fruits of seven new sweet cherry cultivars in terms of their quality traits, bioactive compounds, and nutraceutical potential. The most important characteristics from a production point of view were evaluated to assess the commercial attractiveness of the new cultivars. Moreover, profiles of the three main classes of phenolic compounds and their in vitro antioxidant activity were investigated with the aim of selecting the most promising accessions in terms of health-promoting characteristics. In vitro analyses were then carried out to evaluate the potential neuroprotective activity of the selected cultivars in a differentiated neuron-like SH-SY5Y cell line, focusing on their ability to counteract oxidative stress and modulate BDNF expression.

## 2. Materials and Methods

### 2.1. Plant Material and Sampling Procedure for Fruit Quality Analysis and Phytochemical Determinations

Fruits of sweet cherry (*Prunus avium* L.) cultivars were collected at the full ripeness stage in 2016 from an orchard located in Vignola (Emilia-Romagna, Italy). The study included three reference cultivars (Burlat, Grace Star*, Lapins) and seven new cultivars released from the UNIBO sweet cherry breeding program (Sweet Aryana^®^ PA1UNIBO*, Sweet Lorenz^®^ PA2UNIBO*, Sweet Gabriel^®^ PA3UNIBO*, Sweet Valina^®^ PA4UNIBO*, Sweet Saretta^®^ PA5UNIBO*, and Marysa^®^ PA6UNIBO* grafted on Colt, and Sweet Stephany^®^ PA7UNIBO* grafted on CAB11E). The abbreviated names, Aryana, Lorenz, Gabriel, Valina, Saretta, and Stephany, will be used from here onward.

All cherry trees were grown under similar agronomic techniques (fertilization, irrigation, and pest control). For each cultivar, samples of randomly picked fruits were collected from four different trees of each cultivar. The collected fruits were used either for the determination of standard quality traits or for the gas chromatographic (GC) determination of simple sugars and acids in the juice. The fruit quality analysis was performed on a sample of 30 fruits/plant. The fruit quality parameters analyzed were fruit color (L*, a*, b* measured with a Konica Minolta color CR-400 Chroma Meter, Tokyo, Japan), fruit diameter, fruit weight, delta absorbance (DA) index (using a Cherry DA-meter, Sinteleia, Bologna, Italy), fruit firmness (using a Güss Fruit Texture Analyzer equipped with a 4-mm tip, Strand, South Africa), soluble solids (using an Atago Pocket Refractometer PAL-1, Tokyo, Japan), fruit pH and acidity (using titration with a Crison Titromatic 1S, Barcelona, Spain), and kernel weight. The color coordinates were used to calculate Chroma and Hue angle.

Finally, aliquots from each sample were also immediately frozen in liquid nitrogen, freeze-dried, and stored at −80 °C until extraction as described below.

### 2.2. Analysis of Fruit Quality Traits and GC Determination of Sugars and Acids in Fruit Juices

Analysis of simple sugar and acid contents was performed using a GC approach following the protocol by Bartolozzi et al. [29]. In detail, 500-µL juice aliquots from each sample were added to 450 µL of imidazole buffer (pH 7.0) and stored at 4 °C until analysis. Aliquots of 500 µL of each diluted juice were dried under a continuous air flow prior to derivatization, and 1 µL of the resulting solution was injected into a VARIAN GC 3900 equipped with electronic flow control (EFC), flame ionization detector (FID), CP-Sil 5 CB capillary column (30 m long), and a CP8410 autosampler (helium as carrier gas and nitrogen as make up gas).

### 2.3. Chemicals

Anthocyanins (cyanidin-3-*O*-rutinoside, cyanidin-3-*O*-glucoside, peonidin-3-*O*-glucoside, and peonidin-3-*O*-rutinoside), phenolic acids (chlorogenic acid, neochlorogenic acid, and coumaroylquinic acid), and flavonoids (quercetin-3-*O*-galactoside, kaempferol, kaempferol-3-*O*-rutinoside, quercetin, quercetin-3-*O*-glucoside, quercetin-3-*O*-rhamnoside, and quercetin-3-*O*-rutinoside) pure standards (≥99.5% purity) in powder form; HPLC-grade methanol, acetonitrile, acetone, diethyl ether, ethyl acetate, and water; phosphoric acid (85–87%, *w/w*), hydrochloric acid (37%, *w/w*), monobasic sodium phosphate (≥98%), and sodium hydroxide beads (≥98%); Dulbecco’s modified Eagle’s medium (DMEM), penicillin/streptomycin, 3-(4,5-dimethylthiazol-2-yl)-2,5-diphenyltetrazolium bromide (MTT), 2′,7′-dichlorodihydrofluorescein diacetate (DCFH-DA), monochlorobimane (MCB), all-*trans*-retinoic acid (RA), L-glutamine solution, trypsin-EDTA solution, sodium pyruvate, H_2_O_2_, phosphate buffered saline (PBS), fetal bovine serum (FBS), primers for real-time polymerase chain reaction (RT-PCR), dimethyl sulfoxide (DMSO) and all other chemicals of the highest analytical grade were produced by Merck Italia (Milan, Italy). RNeasy Mini Kit was from Qiagen (Hilden, Germany). Stock solutions (1 mg/mL) of the analytes were prepared by dissolving 10 mg of each pure substance in 10 mL of methanol. Standard solutions were obtained by diluting stock solutions with the mobile phase (initial composition). When stored at −20 °C in the dark, stock solutions were stable for at least one month (as assessed using HPLC); standard solutions were prepared fresh every day.

An XS Instrument (Carpi, Italy) pH 50 pHmeter, a Thermo Fisher Scientific (Carlsbad, CA, USA) CL10 centrifuge, an IKA (Staufen, Germany) A11 Basic knife mill, an IKA Ultra Turrax T-18 homogenizer and an Alpha 1–4 LD (Martin Christ, Osterode Am Harz, Germany) freeze-dryer were used.

### 2.4. Extraction Procedures

After freeze-drying, pits were discarded and the pulp from cherries picked from the same tree was mixed together and finely ground in a knife mill for 4 × 30 s periods. Pulp powder was then subjected to the “coning and quartering” sampling procedure and two technical replicates were carried out.

Flavonoids and anthocyanins were extracted from samples according to the protocol by Ballistreri et al. [30], with modifications. A 1-g aliquot of freeze-dried powder was thoroughly mixed with 5 mL of methanol/HCl (95.5/0.5, *v/v*) mixture and homogenized for 1 min (speed 5.5). The suspension was sonicated for 20 min at 35 °C. After centrifugation for 10 min at 1400× *g*, the supernatant was transferred into another vial and the sediment was extracted twice again with the same procedure. The supernatants were merged, mixed and filtered through Grade 44 (3 µm) ashless filter paper. The final volume of limpid liquid obtained was recorded. The extract was filtered again through a syringe filter (nylon, 0.22 µm pore diameter) from Thermo Fisher Scientific.

Phenolic acids were extracted from samples according to the protocol by Milinović et al. [31], with modifications. A 1-g aliquot of powder was thoroughly mixed with 20 mL of methanol/water (80/20, *v/v*) mixture and homogenized for 1 min (speed 5.5). After centrifugation for 15 min at 1400× *g*, the supernatant was filtered through Grade 44 (3 µm) ashless filter paper. The limpid liquid was brought to 25 mL with 85% methanol in a volumetric flask.

### 2.5. HPLC Determination of Phenolic Compounds

The extracts were injected into a Jasco (Tokyo, Japan) HPLC-DAD system, which consisted of a PU-4180 pump, an MD-4015 PDA detector and an AS-4050 autosampler. The stationary phase was an Agilent (Santa Clara, CA, USA) Zorbax Eclipse Plus C18 reversed-phase column (100 mm × 3 mm I.D., 3.5 μm). The injection volume was 20 µL for all determinations.

The method for flavonoid analysis was adapted from Milinović et al. [31]. Elution was carried out with a mixture of solvent A (water/formic acid 95/5, *v/v*) and solvent B (acetonitrile), with a composition gradient ranging from 97 to 36% of solvent A and flowing at 0.5 mL/min. A signal at 370 nm was used for quantitative purposes. Recovery values of flavonoids in spiked samples ranged from 79.2 to 91.4% (RSD < 9.4%, n = 6).

The chromatographic method for the analysis of phenolic acids was carried out as previously described [32]. Gradient elution was carried out with a mixture of solvent A (50 mM phosphate buffer, pH 2.5) and solvent B (acetonitrile) flowing at 0.7 mL/min, going from 97 to 50% (*v/v*) of solvent A. Signals at 254, 280, and 329 nm were used for analyte quantitation. Recovery values of phenolic acids in spiked samples ranged from 80.2 to 91.3% (RSD < 10.7%, n = 6).

The method for anthocyanin analysis was adapted from Ballistreri et al. [30]. Elution was carried out with a mixture of solvent A (water/formic acid/acetonitrile 87/10/3, *v/v/v*) and solvent B (water/formic acid/acetonitrile 40/10/50, *v/v/v*), with a composition gradient ranging from 94 to 40% of solvent A and flowing at 1.0 mL/min. A signal at 520 nm was used for quantitative purposes. Recovery values of anthocyanins in spiked samples ranged from 78.6 to 89.3% (RSD < 9.7%, n = 6).

### 2.6. In Vitro Antioxidant Activity Assays

2,2′-diphenyl-1-picrylhydrazide (DPPH) assays were carried out on phenolic acid sample extracts (see above) using a Jasco V-630 double beam spectrophotometer, as described elsewhere [33]. For the setup of calibration curves, 950 µL of 11 µM DPPH in methanol and 50 µL of methanolic Trolox (Tx) solution at different concentrations (0.05–2.00 mM), or 50 µL of methanol (blank solution), were thoroughly mixed in a 2-mL polypropylene vial. For sample analysis, the Trolox solution was replaced with 50 µL of suitably diluted sample. The vial was incubated in the dark at room temperature for 24 h, then the absorbance of the solution was read at 515 nm. Calibration curves were set up plotting the discoloration ratio (i.e., [ABS_without TX_/ABS_with TX_] − 1) as a function of Tx concentration. Trolox equivalents (TE) of the samples were calculated interpolating on the calibration curve.

Oxygen radical absorbance capacity (ORAC) assays were carried out on the same extracts (see above) using a Perkin Elmer (Turku, Finland) Viktor X3 multilabel plate reader, essentially as described by Ou et al. [34]. TE were calculated from the relative area under the curve of the emission intensity vs. time plot.

### 2.7. Cell Culture and Viability

The SH-SY5Y human neuroblastoma cell line was obtained from Merck Italia. Cells were grown in DMEM supplemented with 10% (*v/v*) of FBS, 2 mM of L-glutamine, 50 U/mL of penicillin, and 50 μg/mL of streptomycin and maintained at 37 °C in a humidified incubator with 5% CO_2_, as previously reported [35]. Cells were used for experiments after inducing their differentiation with RA (10 μM) for 7 days.

To test viability, cells were treated with different concentrations (0.1–100 µg/mL) of five cherry extracts (Burlat, Grace Star, Gabriel, Lorenz, Marysa) for 24 h. Oxidative stress was induced with 700 µM H_2_O_2_ for 1 h. Cell viability was evaluated by measuring MTT reduction as previously reported [36]. Briefly, at the end of the experiments, MTT was added to the medium (final concentration 0.5 mg/mL) and incubated for 90 min at 37 °C. After incubation, MTT solutions were removed, DMSO was added, and the absorbance was measured using a Viktor X3 multilabel plate reader at a wavelength of 595 nm.

### 2.8. Intracellular ROS Measurement

The formation of intracellular ROS was evaluated using the fluorescent DCFH-DA probe as previously reported [37]. Briefly, SH-SY5Y cells were treated with 50 µg/mL of each cherry extract for 24 h, then incubated with 10 µM DCFH-DA in DMEM 1% FBS w/o phenol red for 30 min. After removal of DCFH-DA, cells were incubated with 400 µM H_2_O_2_ in DMEM 1% FBS w/o phenol red for 15 min. Then, H_2_O_2_ was removed and replaced using PBS. Cell fluorescence was measured at 485 nm (excitation) and 535 nm (emission) with a Viktor X3 multilabel plate reader.

### 2.9. Determination of Reduced Glutathione (GSH) Levels

Reduced glutathione (GSH) levels were determined using the monochlorobimane (MCB) fluorometric assay as previously reported [38]. Briefly, SH-SY5Y cells were treated with 50 µg/mL of each cherry extract for 24 h before the induction of oxidative stress with 700 µM H_2_O_2_ for 1 h. After treatment, cells were incubated with 50 µM MCB 1% FBS w/o phenol red for 30 min at 37 °C. After incubation, fluorescence was measured at 355 nm (excitation) and 460 nm (emission) with a Viktor X3 multilabel plate reader.

### 2.10. Analysis of mRNA Expression

SH-SY5Y cells were treated with 50 µg/mL of each cherry extract for 24 h and, after treatment, total RNA was extracted using an RNeasy Mini Kit, following the manufacturer’s protocol. The yield and purity of RNA were measured using a NanoVue spectrophotometer (GE Healthcare, Milan, Italy).

mRNA was reverse-transcribed into cDNA starting from 1 µg of total RNA using an iScript cDNA Synthesis Kit (BIO-RAD, Hercules, CA, USA), following the manufacturer’s protocol. The subsequent PCR was performed in a total volume of 10 µL containing 2.5 µL (12.5 ng) of cDNA, 5 µL SsoAdvanced Universal SYBR Green Supermix (BIO-RAD), and 0.5 µL (250 nM) of each primer. Primers used were: BDNF, 5′CAAAAGTGGAGAACATTTGC3′ (forward) 5′AACTCCAGTCAATAGGTCAG3′ (reverse); glutathione reductase (GR), 5′GACCTATTCAACGAGCTTTAC3′ (forward) 5′CAACCACCTTTTCTTCCTTG3′ (reverse); NAD(P)H quinone oxidoreductase (NQO1), 5′AGTATCCACAATAGCTGACG3′ (forward) 5′TTTGTGGGTCTGTAGAAATG3′ (reverse); and ribosomal protein S18 (RPS18), 5′CAGAAGGATGTAAAGGATGG3′ (forward) 5′TATTTCTTCTTGGACACACC3′ (reverse) from Merck Italia. RPS18 was used as a reference gene. cDNA amplification was started by activating the polymerase for 30 s at 95 °C, followed by 40 cycles of 5 s at 95 °C and 30 s at 60 °C. A melt curve was run to ensure quality control and generation of a single product. Normalized expression levels were calculated relative to control cells according to the 2^−^^ΔΔCT^ method [39].

### 2.11. Statistical Analysis

Each experiment was performed at least three times, and all values are represented as mean ± standard error median (SEM). One-way analysis of variance (ANOVA) was used to compare differences among groups, followed by Tukey’s test or Dunnett’s test (Prism 6; GraphPad Prism Software, San Diego, CA, USA). Values of *p* < 0.05 were considered statistically significant. Correlation analysis was carried out with the same software.

A canonical discriminant analysis of some collected data (ORAC values, anthocyanin, phenolic acid and flavonol contents) was performed with the library ‘candisc’ of the R software (3.6.1 version). Correlations among different fruit traits and biochemical variables were calculated using Statistica 12 software.

## 3. Results

### 3.1. Physical-Chemical Characteristics of Sweet Cherry Cultivars

The selected set of cultivars uniformly covers the sweet cherry harvesting season in the Emilia-Romagna region, starting from Burlat (May 23rd) and ending with Stephany (June 17th). The complete fruit quality description of each cultivar is reported in the Supporting Information materials. In general, significant differences among cultivars were identified for all the measured quality traits. The new varieties from the ‘Sweet’ series produce large fruits, with a caliper ranging from about 28 mm (Aryana) to more than 32 mm (Stephany and Valina; Figure S1). As expected, early varieties (Burlat, Aryana, Marysa, and Lorenz) bear fruits slightly smaller than those of late cultivars (Stephany and Valina), whose fruits reached weights of more than 17 g each. As expected, there is a high correlation (0.78–0.80; Table S1) between fruit and kernel sizes, and Aryana and Marysa produced the fruits with the smallest kernels.

Regarding the red color at maturity, fruits of Lapins, Marysa, and Valina showed higher values of a*, b*, and chroma (bright red) compared to those of Gabriel, Lorenz, and Stephany (dark red; Figure S2). The DA harvesting index results were strongly negatively correlated with some fruit color parameters like a* and chroma (−0.90; Table S1); therefore, the highest DA indexes were recorded on the dark red fruiting cultivars, having low values of either a* or chroma.

As concerns soluble solids, the lowest values were recorded in Lapins, Marysa, and Burlat, while the highest ones in Lorenz, Stephany and Gabriel (Figure S3). Regarding titratable acidity, Burlat, Lapins, and Aryana were the least acidic fruits, while Gabriel and Stephany were the most acidic ones. The acidity of the last cultivar was very high (12.22 mg/L) in comparison with all the others, but the global taste was well balanced, because the soluble solid content was also the highest (19.47°Brix).

The GC analysis of the fruit juices revealed that malic acid represented more than 93% of total acid content in Gabriel, Grace Star, Valina, Stephany, and Lapins (Figure S4). Burlat, Aryana, and Lorenz contained less than 90% of malic acid and around 7–8% of succinic acid. Quinic acid was the third acid, in order of importance, in cherry fruits, but its content only ranged between 1.9 and 3.6% of the total. A high correlation (0.86) was found between titratable acidity and malic acid content using GC (Table S2).

Regarding monosaccharides and analogues, their content is variable among cultivars; glucose and fructose represented the most important sugars of cherry juices (46.5 and 41.0%, respectively), followed by sorbitol, while others (sucrose, inositol or xylose) were present only in trace amounts (Figure S5). Glucose content ranged from 44% (Saretta and Stephany) to 47–48% (Marysa, Burlat and Aryana). The highest fructose percentage was found in Burlat and Gabriel (43–44%) and the lowest in Saretta and Stephany (about 39%). A high correlation (0.98) was observed between fructose and glucose contents, while correlation with total soluble solid content was much lower (0.41; Table S3).

### 3.2. Phenolic Compound Profile

The characterization of new cultivars for their phenolic profile was carried out and compared to that of the three reference cultivars Burlat, Grace Star, and Lapins.

The main classes of phenolic compounds in the pulp of sweet cherry are anthocyanins, phenolic acids (hydroxycinnamic acid derivatives), and flavonols; thus, HPLC-DAD analyses were carried out to identify and quantify compounds belonging to these three classes of secondary metabolites.

**Figure 1:**
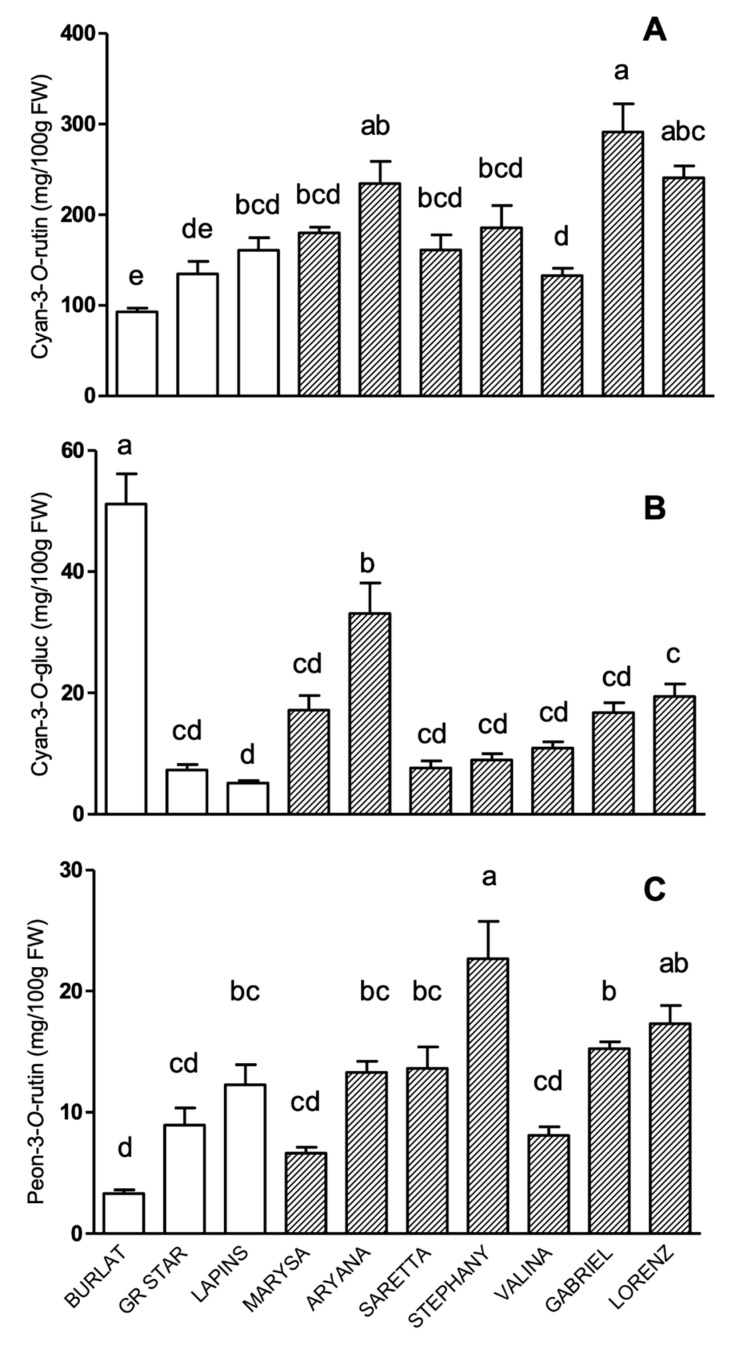
Anthocyanins levels (mg/100 g FW) in sweet cherry cultivars: (**A**) cyanidin-3-*O*-rutinoside, (**B**) cyanidin-3-*O*-glucoside, and (**C**) peonidin-3-*O*-rutinoside. Data are the mean ± SEM of four biological replicates. Different letters indicate statistical significance (*p* < 0.05).

As concerns anthocyanins, in all the genotypes cyanidin-3-*O*-rutinoside was by far the most abundant one, ranging from 92 to more than 300 mg/100 g of fresh weight (FW), followed by cyanidin-3-*O*-glucoside, and peonidin-3-*O*-rutinoside, respectively (Figure 1). Peonidin-3-*O*-glucoside was also detected in all cultivars, but at levels below 1 mg/100 g FW (data not shown).

Among new cultivars, Gabriel, Lorenz, and Aryana were the richest in cyanidin-3-*O*-rutinoside (Figure 1A), and in particular, Gabriel had levels about twice those of the three reference cultivars. All other new accessions showed cyanidin-3-*O*-rutinoside levels rather similar among them, and significantly higher than those of Burlat.

As concerns cyanidin-3-*O*-glucoside (Figure 1B), the highest levels were found in Burlat, which showed a concentration about seven times higher than the other reference genotypes. Among new accessions, Aryana showed the highest level, while all the others did not differ significantly among each other and compared to Grace Star and Lapins.

As concerns peonidin-3-*O*-rutinoside levels (Figure 1C), cultivars can be divided into three groups: the richest one was Stephany, with more than 20 mg/100 g FW, followed by Lorenz, Gabriel, Saretta, Aryana, and Lapins, with levels ranging from 10 to 20 mg/100 g FW; the group with the lowest levels (less than 10 mg/100 g FW) was represented by Valina, Marysa, Grace Star, and Burlat.

**Figure 2:**
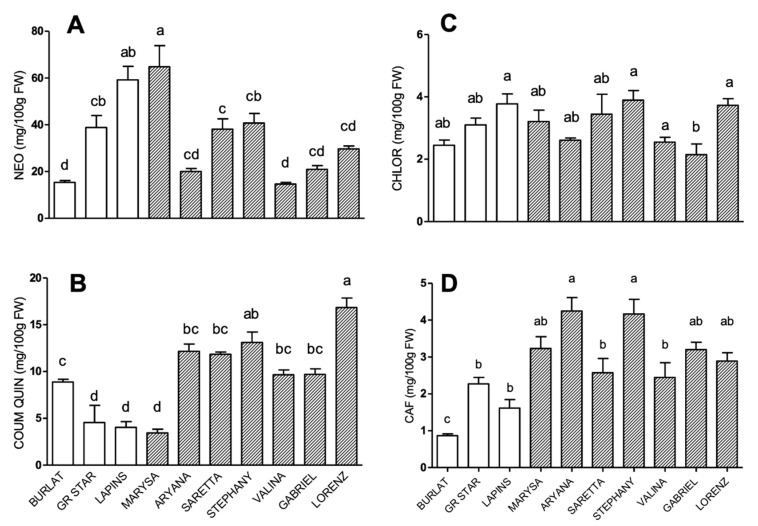
Phenolic acid levels (mg/100 g FW) in sweet cherry cultivars: (**A**) neochlorogenic acid, (**B**) coumaroylquinic acid, (**C**) chlorogenic acid, and (**D**) caffeic acid. Data are the mean ± SEM of four biological replicates. Different letters indicate statistical significance (*p* < 0.05).

Phenolic acid profiles are shown in Figure 2. In all accessions, neochlorogenic acid was the main hydroxycinnamic acid derivative, followed by coumaroylquinic, chlorogenic, and caffeic acids, respectively.

Neochlorogenic acid levels ranged from 15 to 65 mg/100 g FW (Figure 2A). Within this range, three groups could be clearly defined: the high-level group, comprising Marysa and Lapins, with levels higher than 50 mg/100 g FW; the intermediate group, comprising Grace Star, Saretta, and Stephany, with levels ranging from 30 to 50 mg/100 g FW, and the low-level group, comprising Burlat, Aryana, Valina, Gabriel, and Lorenz, with levels below 30 mg/100 g FW.

Coumaroylquinic acid content ranged from 1.5 to 17 mg/100 g FW. Lorenz had the highest and Marysa the lowest coumaroylquinic acid levels (Figure 2B). Chlorogenic and caffeic acid content ranged from 1 to 4 mg/100 g FW in all cultivars (Figure 2C,D), and slight fluctuations were observed among them. For caffeic acid, all new cultivars showed levels significantly higher than Burlat, and among them, Aryana, Stephany, Marysa, Gabriel, and Lorenz were the richest ones (Figure 2D).

Within the class of flavonols, quercetin-3-*O*-rutinoside (rutin) was the most abundant compound. Quercetin-3-*O*-glucoside and kaempferol-3-*O*-rutinoside were also detected in all cultivars, but at very low levels, in the 0.02–1.0 mg/100 g FW range for the former and 0.08–1.2 mg/100 g FW for the latter (data not shown).

**Figure 3:**
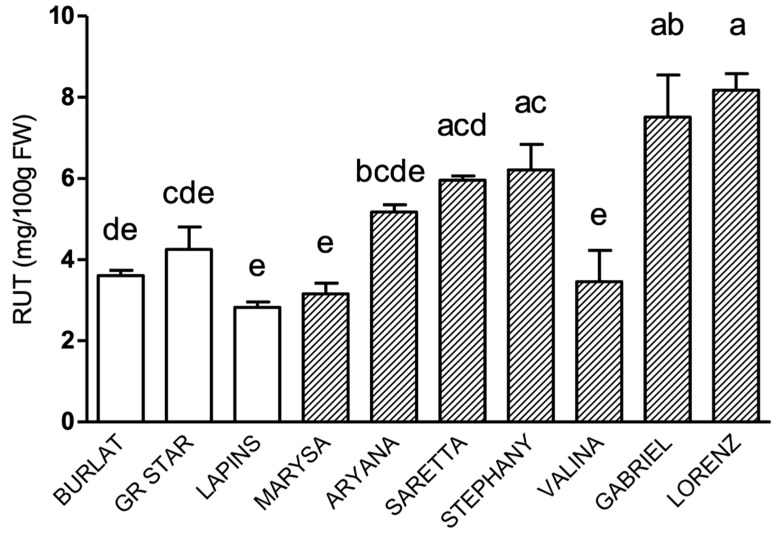
Rutin levels (mg/100 g FW) in sweet cherry cultivars. Data are the mean ± SE of four biological replicates. Different letters indicate statistical significance (*p* < 0.05).

Rutin patterns in all genotypes are shown in Figure 3. Lorenz and Gabriel had the highest rutin levels among all genotypes, significantly higher compared to all reference cultivars.

### 3.3. Total Anthocyanin Index, Total Phenolic Acid Index, and In Vitro Antioxidant Activity

Total anthocyanin index (TAI) and total phenolic acid index (TPAI), which were calculated as the sum of all individual concentrations, are shown in Figure S6. The pattern was very similar to that of the major components of each class, namely cyanidin-3-*O*-rutinoside and neochlorogenic acid, respectively.

**Figure 4:**
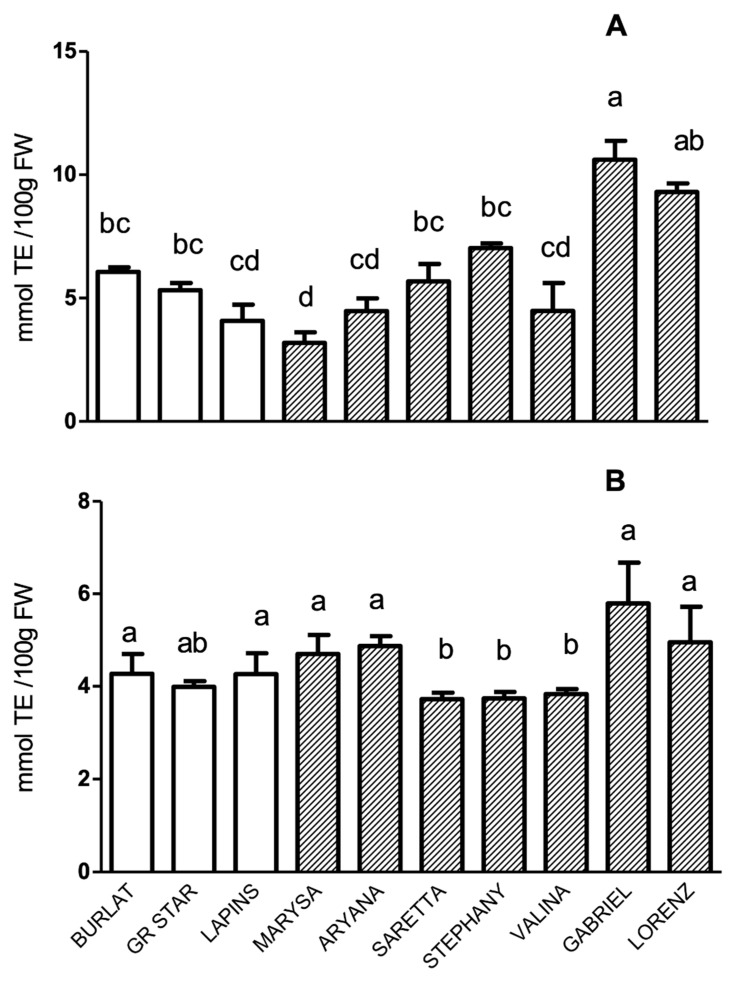
Antioxidant activity (mmol Trolox equivalents/100 g FW) of sweet cherry cultivars, assayed using (**A**) the DPPH and (**B**) the ORAC assays.

The antioxidant activity (AA) of fruit methanolic extracts was measured using the DPPH and ORAC assays (Figure 4). The pattern of AA, according to the DPPH assay, showed that Gabriel was the most powerful antioxidant accession, reaching levels of about 10 mmol TE/100 g FW (Figure 4A). As concerns AA results measured using the ORAC assay, differences among genotypes were much less pronounced in comparison to those of DPPH, and differences observed between new and reference cultivars were not significant (Figure 4B). Nevertheless, a good correlation (0.53) was found between ORAC-assayed AA and TAI (Table S4).

**Figure 5:**
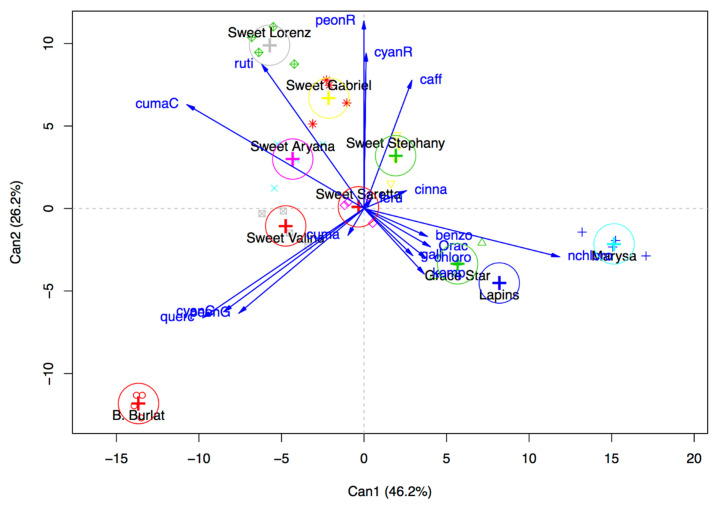
Canonical discriminant analysis of the AA (ORAC) and anthocyanin, phenolic acid, and flavonol contents of sweet cherry extracts. Can1 = canonical component 1; Can2 = canonical component 2.

The results of the canonical discriminant analysis of the AA (ORAC), and anthocyanin, phenolic acid, and flavonol contents is shown in Figure 5. The first two components explain around 73% (46.2 and 26.2) of the observed variability. The first component is significantly related to the content of neochlorogenic acid (+0.83), while the second one is related to peonidin-3-*O*-rutinoside (+0.80) and cyanidin-3-*O*-rutinoside (+0.66) contents. Based on this explorative analysis, two reference cultivars (Burlat and Grace Star) and three new cultivars (Gabriel, Lorenz and Marysa) resulted to be very diverse in respect to the contents of phenolic compounds and were selected for the subsequent evaluation of the protective effects of cherry extracts.

### 3.4. Neuroprotective Effect of Cherry Extracts against Oxidative Stress

**Figure 6:**
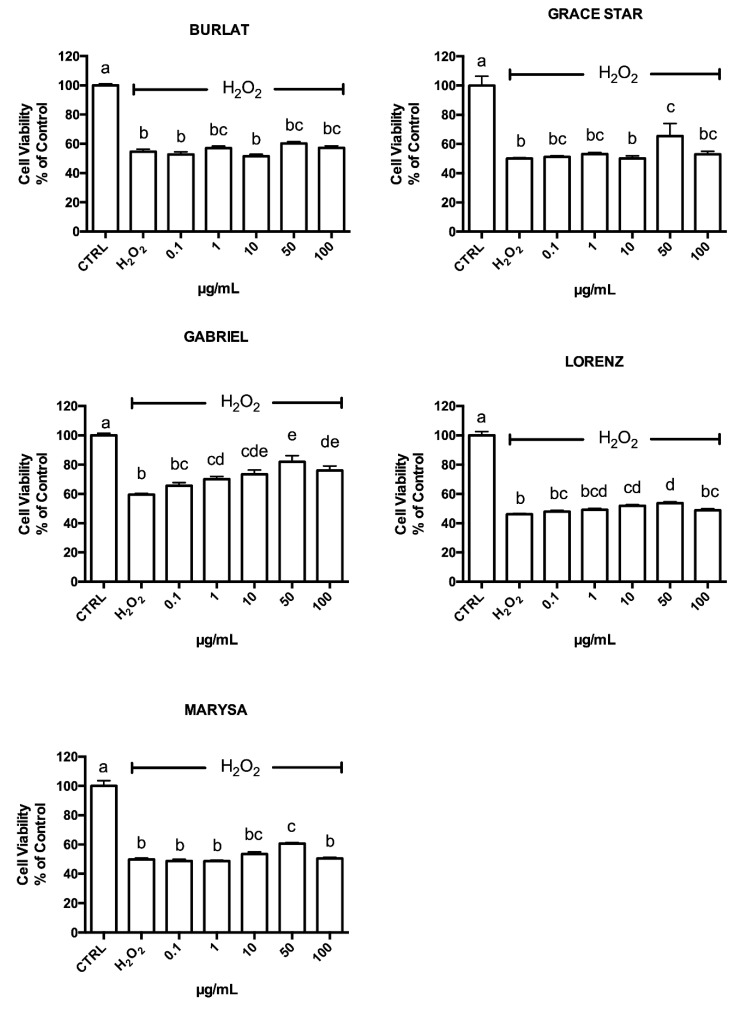
Protective effect of cherry extracts against H_2_O_2_-induced damage (700 µM) to SH-SY5Y cells. Each bar represents the mean ± SEM of at least three independent cell viability experiments. Different letters indicate statistical significance (*p* < 0.05).

The cytotoxicity of the different cherry extracts was evaluated using the MTT assay, treating differentiated SH-SY5Y cells with the extracts (0.1–100 μg/mL) for 24 h (Figure S7). No cytotoxicity was observed, as cell viability was not influenced by the exposure to extracts up to 100 μg/mL. In order to assess the potential protective role of the tested extracts, cells were pretreated with 0.1–100 μg/mL of each extract for 24 h before exposing them to H_2_O_2_ for 1 h (Figure 6) to induce oxidative stress as previously reported [35]. Gabriel, Lorenz, Grace Star, and Marysa cultivars were able to significantly increase viability in cells exposed to H_2_O_2_. In particular, Gabriel was the most effective one, with significant protection observed at all concentrations higher than 1 µg/mL, while Lorenz produced significant protection at two concentrations (10 and 50 µg/mL). Burlat did not show any protection against oxidative stress.

**Figure 7:**
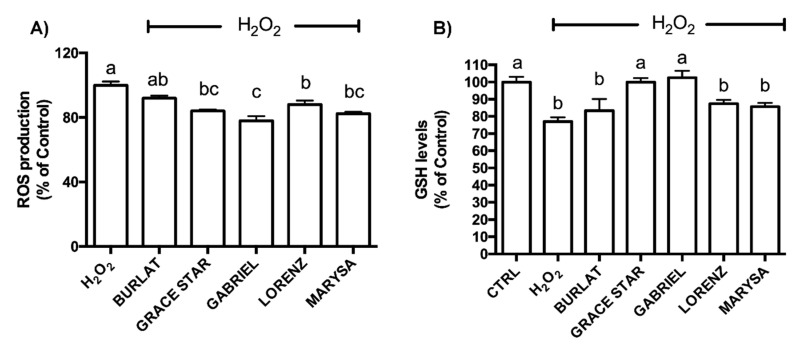
Effects of cherry extracts on intracellular (**A**) reactive oxygen species (ROS) and (**B**) reduced glutathione (GSH) levels in SH-SY5Y cells. ROS data are expressed as a percentage compared to H_2_O_2_-treated cells, and GSH levels are expressed as a percentage compared to control (CTRL) cells; each bar represents the mean ± SEM of at least three independent experiments. Different letters indicate statistical significance (*p* < 0.05).

As 50 μg/mL was the only concentration that led to significant protection in most extracts, this concentration was used for subsequent experiments. To better characterize the protection mechanisms, the ability of the cherry extracts to counteract H_2_O_2_-induced intracellular ROS production was investigated using the DCFH-DA assay. As reported in Figure 7A, in agreement with viability data, Gabriel, Grace Star, Marysa, and Lorenz led to a significant reduction in intracellular ROS levels in cells exposed to H_2_O_2_, while Burlat did not produce any significant effect.

Since GSH is the most abundant intracellular antioxidant, the effect of the cherry extracts on intracellular GSH levels was evaluated using the MCB assay. SH-SY5Y cells were treated with 50 μg/mL of each extract for 24 h and then exposed to H_2_O_2_ (Figure 7B). As expected, H_2_O_2_ treatment led to significant reduction of GSH levels in respect to control cells. Grace Star and Gabriel extracts significantly increased GSH levels when compared to H_2_O_2_-exposed cells, up to values comparable to those of control cells. Marysa, Lorenz, and Burlat did not increase GSH levels compared to peroxide.

### 3.5. Effect of Cherry Extracts on Antioxidant Enzyme and BDNF Expression

**Figure 8:**
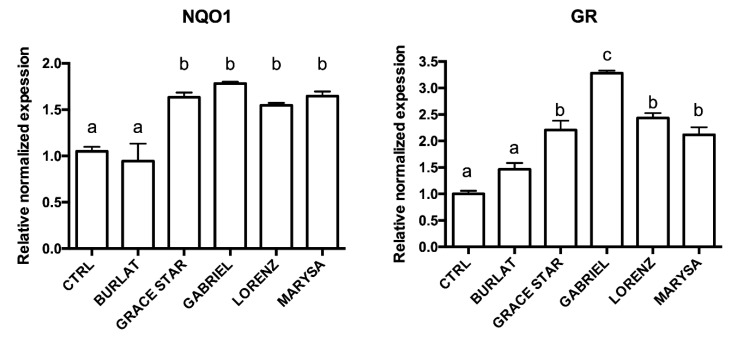
Effect of cherry extracts on the expression of NAD(P)H quinone oxidoreductase (NQO1) and glutathione reductase (GR) in SH-SY5Y cells. Each bar represents the mean ± SEM of at least three independent experiments. Different letters indicate statistical significance (*p* < 0.05).

The cherry extracts could act through the modulation of the endogenous antioxidant system, involved in the formation of GSH and hydroquinones in the cells. To evaluate this possibility, the expression of two endogenous antioxidant enzymes, namely GR and NQO1, was analyzed using RT-PCR (Figure 8). Cells were treated with 50 μg/mL of the five extracts before measuring changes in mRNA expression. Treatment with extracts from Grace Star, Gabriel, Lorenz, and Marysa cultivars was able to significantly up-regulate NQO1 and GR, while Burlat did not influence the expression of these enzymes. Gabriel was the most effective one in up-regulating GR. Interestingly, a positive correlation was found between GR expression and TAI (0.89, data not shown).

**Figure 9:**
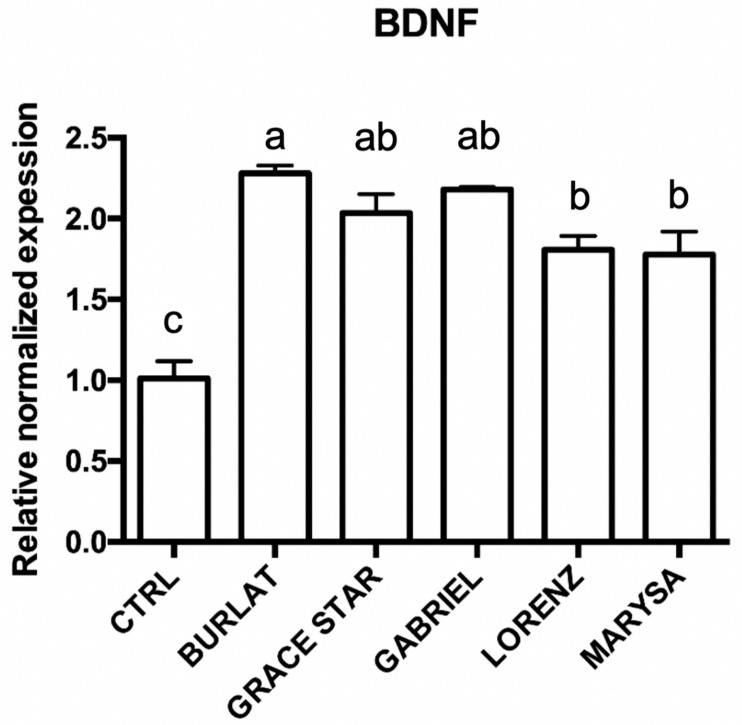
Effect of cherry extracts on the expression of brain-derived neurotrophic factor (BDNF) in SH-SY5Y cells. Each bar represents the mean ± SEM of at least three independent experiments. Different letters indicate statistical significance (*p* < 0.05).

**Figure 10:**
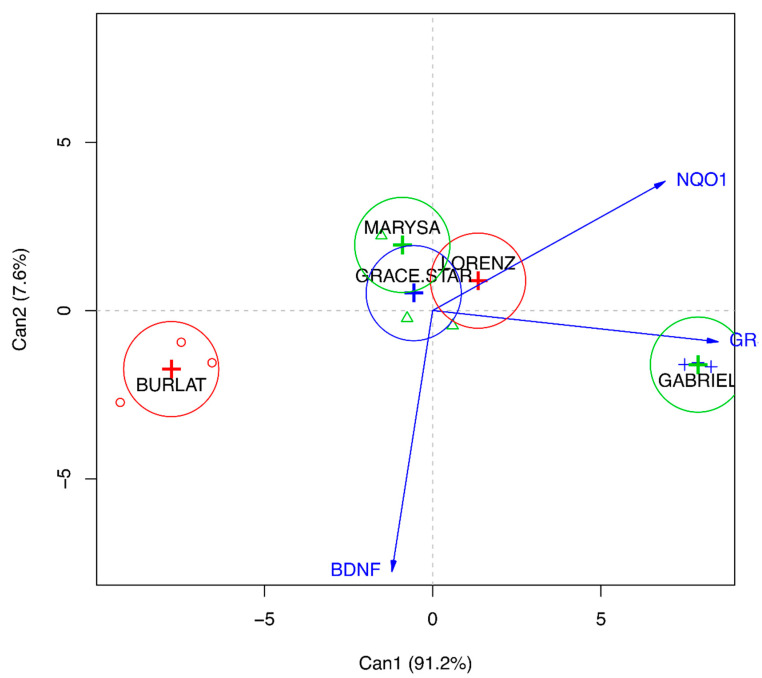
Canonical discriminant analysis of BDNF, GR and NQO1 gene expression after treatment with sweet cherry extracts. Can1 = canonical component 1; Can2 = canonical component 2.

The levels of brain-derived neurotrophic factor (BDNF) are strongly reduced in different neurodegenerative diseases [27]. For this reason, the effect of the different extracts on BDNF expression levels was investigated. Cells were treated with 50 μg/mL of the extracts before measuring changes in BDNF mRNA expression. Interestingly, all the extracts were able to significantly up-regulate BDNF in comparison to control cells (Figure 9), suggesting a protective role of cherries in neurodegeneration, possibly through a mechanism other than the antioxidant activity. To better understand the relationship between the global effect of the different extracts on these proteins and neuroprotection, a canonical discriminant analysis was conducted (Figure 10). Data evidenced that Gabriel is the cultivar with the highest neuroprotective activity, and this seems to be related to the up-regulation of GR; in fact, the first component explains 91.2% of the variability, and this is mainly due to GR. On the other hand, Burlat falls in the opposite part of the graph underlining its lower impact on these proteins and its lower neuroprotective activity.

## 4. Discussion

Results here obtained show that the cherry cultivars clearly differ from each other in fruit quality parameters and in health-promoting compound content. Indeed, the pattern of polyphenolic compounds known to characterize *Prunus avium* fruits showed significant quantitative differences among cultivars.

Fruit quality traits, as well as health-promoting compounds, are known to be strongly influenced by the environment, orchard management [40], and harvesting date [5], but a rather wide genetic variability among the selected cultivars has been observed for the compounds analyzed in this study.

The main quality traits of the new cultivars confirmed their overall excellent organoleptic attributes compared to reference cultivars, such as for weight, firmness, color, and sugar/acidity ratio [5,41]. One of the main peculiarities of these new cultivars is their different ripening times, which collectively cover a time period from the middle of May to the end of June, thus allowing uniform coverage of the very short harvest season for sweet cherries.

Sugar and acid composition, as well as flesh firmness, are known to be key factors in determining the fruit quality and taste of sweet cherries. In general, sugar and acid composition is in line with those reviewed by Chockchaisawasdee et al. [42]. The correlation between total sugars and soluble solids is significant but not very high (0.44, data not shown), but it is known that the Brix value is affected also by other compounds such as acids and amino acids [42], as confirmed by the observed significant positive correlation (0.52, data not shown) between soluble solids and fruit acidity.

Glucose and fructose represent the main sugars in sweet cherries, as reported elsewhere [43]. These two sugars are reported to account for about 90% of the total sugars in cherry fruits [42]. The total amount of these two sugars and their relative amounts are essential in determining the overall sweetness of the fruits, since the sweetness power of fructose is known to be higher than that of glucose [44].

The qualitative profile of hydroxycinnamic acid derivatives in all cultivars analyzed here reflects that reported by Picariello et al. for sweet cherries [45], with neochlorogenic, coumaroylquinic, and chlorogenic acids as main compounds. According to these authors, the relative content of hydroxycinnamates greatly varies depending on genotypes. In all cultivars here analyzed, neochlorogenic acid represents the predominant compound, and this agrees with results reported by other authors [43,46,47], although a different ratio between neochlorogenic and coumaroylquinic or chlorogenic acid has also been observed in some cases [30,48,49]. The levels of these colorless polyphenols found in the new cultivars were very similar to those reported by some authors in other cultivars [46]; however, higher or lower concentrations have been reported elsewhere as well [43,49,50]. These quantitative fluctuations are expected, since they are related to several factors, including genotype, growing conditions, ripeness stage, post-harvest storage, sample preparation, extraction procedures, and analytical techniques.

Results concerning anthocyanin profiles are consistent with most of the results reported in the literature, which identify cyanidin-3-*O*-rutinoside, cyanidin-3-*O*-glucoside, and peonidin-3-*O*-rutinoside as the major anthocyanins and peonidin-3-*O*-glucoside as a minor component [11,46,51]. The concentrations of cyanidin-3-*O*-rutinoside in all cultivars here analyzed ranged between 92.7 and 291.3 mg/100 g FW. These values are similar to those found by Nawirska-Olszaňska et al. [51] in old cherry cultivars grown in the Czech Republic, but were significantly higher than those reported by other authors; Ballistreri et al. [30] found levels of cyanidin-3-*O*-rutinoside ranging from 5.69 to 68.28 mg/100 g FW in twenty-four cultivars grown in Sicily (Italy), and Mozetič et al. [52] reported levels between 28.2 and 62.1 mg/100 g FW in five Slovenian cultivars. Even lower levels (between 1.09 and 14.8 mg/100 g FW) were reported by Usenik et al. [43] in different accessions grown in Slovenia. Besides the numerous factors known to influence the phenolic compound composition and concentrations in fruits, these differences can also be due to the fact that fresh fruits were utilized as the starting material in most reported studies, while lyophilized fruits were used here, and this could provide, e.g., higher extraction yields. Anthocyanin content was shown to be correlated with color parameters scored on fresh fruits. The higher the content of anthocyanins, mainly cyanidin-3-*O*-rutinoside and -glucoside, the darker the fruit color was, as expressed by lower L* and a* values (negative correlation of −0.53 and −0.47 against TAI, Table S4). The TAI of dark red fruits (Lorenz, Gabriel, and Stephany) was twice that of bright red fruits (Burlat).

The AAs of cherry extracts, measured using two widely used assays, were not univocal and different trends were observed between DPPH and ORAC. This is not surprising, since these assays are very different in their underlying chemical reaction and measurement procedures, and discrepancies in the AA trend measured using different assays have been reported. According to Ou et al. [53], who carried out one of the most comprehensive AA studies to date on 927 vegetables, the ORAC method was considered more chemically relevant to chain-breaking AA, while the stable DPPH radical bears no similarity to the highly reactive, transient radicals formed in the cells during lipid peroxidation. Our results indicate that a rather good correlation exists between the ORAC-assayed AA and TAI (Table S4), suggesting that this class of phenolic compounds gives a good contribution to the antioxidant capacity.

Taken together, the results presented here clearly indicate that the profile of bioactive polyphenol compounds is strictly dependent on genotype, and as concerns new cultivars, Marysa was the richest cultivar in colorless polyphenols, Gabriel in anthocyanins, and Lorenz in rutin. Compared to reference cultivars, Gabriel had a TAI value two‒three times higher, and Lorenz had a rutin content almost twice higher, while in the case of phenolic acids the results were not so univocal. This indicates that, generally, sweet cherry accessions resulting from the breeding program can be considered as rich in health-promoting compounds as the reference cultivars, or even more so.

The classes of compounds identified in the new cherry cultivars have been reported to exhibit neuroprotective activity [54,55]. Thus, we evaluated the protective effect of the five most diverse cultivars (as assessed using PCA analysis) against oxidative stress in neuron-like differentiated SH-SY5Y cells. These cells are widely used as an in vitro model of neuronal function [56] and their exposure to H_2_O_2_ triggers metabolic modifications leading to cell death and mimicking the events that might occur during neurodegeneration [57]. Interestingly, treatment with the new cherry cultivar extracts protected cells against oxidative stress induced by H_2_O_2_. This effect was associated to a significant reduction in intracellular ROS and a significant increase in GSH levels. In particular, Gabriel, which is characterized by the highest content of anthocyanins, was the most effective one in this regard. It has been indicated that anthocyanins can reach the brain in vivo, as absorbed anthocyanins and their metabolites are able to cross the blood‒brain barrier [58,59]. Our data agree with the results reported by Leong et al. [60], who observed that cherry cultivars richest in anthocyanins were more effective in protecting Caco-2 cells from H_2_O_2_-induced damage compared to other cultivars. On the contrary, another study compared three different extracts of Saco sweet cherry, namely the non-colored fraction, colored fraction, and total extract [61]. The colored fraction was the least effective in protecting Caco-2 cells against oxidative stress induced by tert-butylhydroperoxide. The antioxidant activity of cherry extracts was also observed in neuronal cells. In particular, Matias et al. [62] demonstrated that a phenolic-rich extract from the Saco cherry cultivar, mainly composed of anthocyanins, counteracts H_2_O_2_-induced damage in SK-N-MC cells.

The cultivars that performed better in terms of neuroprotective potential (Gabriel and Lorenz) were especially rich in cyanidin-3-*O*-rutinoside, peonidin-3-*O*-rutinoside, rutin, and some minor phenolic acids (i.e., caffeic acid), while in the Burlat fruits, the same compounds are in much lower amounts. From our data, among anthocyanins, rutinoside forms seem to be more effective than glucoside ones. The observed neuroprotective activity of caffeic acid is in line with recent studies that demonstrated profound effects of this compound in the brain, including protection from toxicity induced by a variety of agents and/or experimental models of Alzheimer’s disease [63].

To better clarify the intracellular antioxidant mechanisms of these extracts we evaluated their ability to modulate two antioxidant enzymes: GR and NQO1. GR is an important part of the cellular antioxidant defense as it maintains the intracellular ratio of reduced/oxidized glutathione (GSH/GSSG) [64]. The important antioxidant activity of NQO1 is related to its catalytic mechanism; in fact, it catalyzes exclusively the two-electron reduction of quinones to their corresponding hydroquinones, thus avoiding the generation of highly reactive semiquinones and ROS [65]. Interestingly, the new cherry cultivars triggered a significant up-regulation of these two enzymes, and Gabriel, the most effective one in counteracting oxidative stress, significantly increased GR expression in respect to the other cultivars, suggesting that the observed neuroprotective effect could mainly be ascribed to the up-regulation of this enzyme. Other authors demonstrated that anthocyanins are able to up-regulate GR and NQO1 [66], but to our knowledge, this is the first time that this up-regulation has been observed after cherry extract treatments. In the brain, BDNF has a key role in survival, maintenance, and regeneration of specific neuronal populations [67]. Depletion of this neurotrophic factor has been associated with different neurodegenerative disorders such as Parkinson’s and Alzheimer’s diseases [26,27] and strategies able to increase its levels are considered of great therapeutic interest. All the tested extracts significantly increased BDNF levels and this seems not to be related to the anthocyanin content as also cultivars with low TAI had a strong effect on BDNF expression. Williams et al. [68] observed that a blueberry extract increased BDNF levels in mouse hippocampus and ascribed this effect not only to anthocyanins but also to flavonols.

## 5. Conclusions

In conclusion, our data suggest that these cherry extracts possess neuroprotective activity in vitro, but in vivo studies should be carried out to assess real bioavailability. In fact, cherries are overwhelmingly consumed as fresh fruit, so the study was focused on their most common use, i.e., food use. This is the reason why FW-based results have been shown throughout. In our opinion, the “take home message” is that some of the studied cherry cultivars possess an added value in terms of bioactive compounds with respect to other cultivars, and we hope that in the future the quality of cherries could be evaluated not only in terms of their organoleptic properties, but also for their nutraceutical potential. Moreover, the precise role of the studied compounds has to be further investigated to understand if their positive effect on neuroprotection is due to the total amounts or to their balance. Nevertheless, the variability observed for these compounds in the analyzed cultivars, especially in the newly bred cultivars, could be a key step in selecting the best parents to be used in future breeding programs and pave the way to further improve the nutraceutical value of future sweet cherry cultivars.

## Supplementary Materials

The supplementary materials are available online at https://www.mdpi.com/2076-3921/9/8/677/s1.
