# Supplementary material for: Fruit Quality Characterization of New Sweet Cherry Cultivars as a Good Source of Bioactive Phenolic Compounds with Antioxidant and Neuroprotective Potential
Source: Antioxidants (Basel). 2020 Jul 28;9(8):677. doi: 10.3390/antiox9080677 (PMC7463759; doi:10.3390/antiox9080677)
Supplement: Supplementary file 1 [file antioxidants-09-00677-s001.pdf]

Supplementary Materials

Fruit quality characterization of new sweet cherry cultivars as a good source of bioactive phenolic compounds with antioxidant and neuroprotective potential

Fabiana Antognoni[1], Giulia Potente[1], Roberto Mandrioli[1,*], Cristina Angeloni[2], Michela Freschi[1], Marco Malaguti[1], Silvana Hrelia[1], Stefano Lugli[3,4], Fabio Gennari[3], Enrico Muzzi[3], Stefano Tartarini[3]

[1] Department for Life Quality Studies, Alma Mater Studiorum – University of Bologna, Corso d'Augusto 237, 47921 Rimini, Italy; fabiana.antognoni@unibo.it; giulia.potente@unibo.it; roberto.mandrioli@unibo.it; michela.freschi2@unibo.it; marco.malaguti@unibo.it; silvana.hrelia@unibo.it

[2] School of Pharmacy, University of Camerino, Via Madonna delle Carceri 9, 62032 Camerino (MC), Italy; cristina.angeloni@unicam.it

[3] Department of Agricultural and Food Science, Alma Mater Studiorum – University of Bologna, Viale Fanin 46, 40127 Bologna, Italy; fabio.gennari3@unibo.it; enrico.muzzi@unibo.it; stefano.tartarini@unibo.it

[4] Department of Life Sciences, University of Modena and Reggio Emilia, Biology Building, Via Giuseppe Campi 213/D, 41125 Modena, Italy; stefano.lugli61@unimore.it

* Correspondence: roberto.mandrioli@unibo.it; Tel.: +39-0541-434624

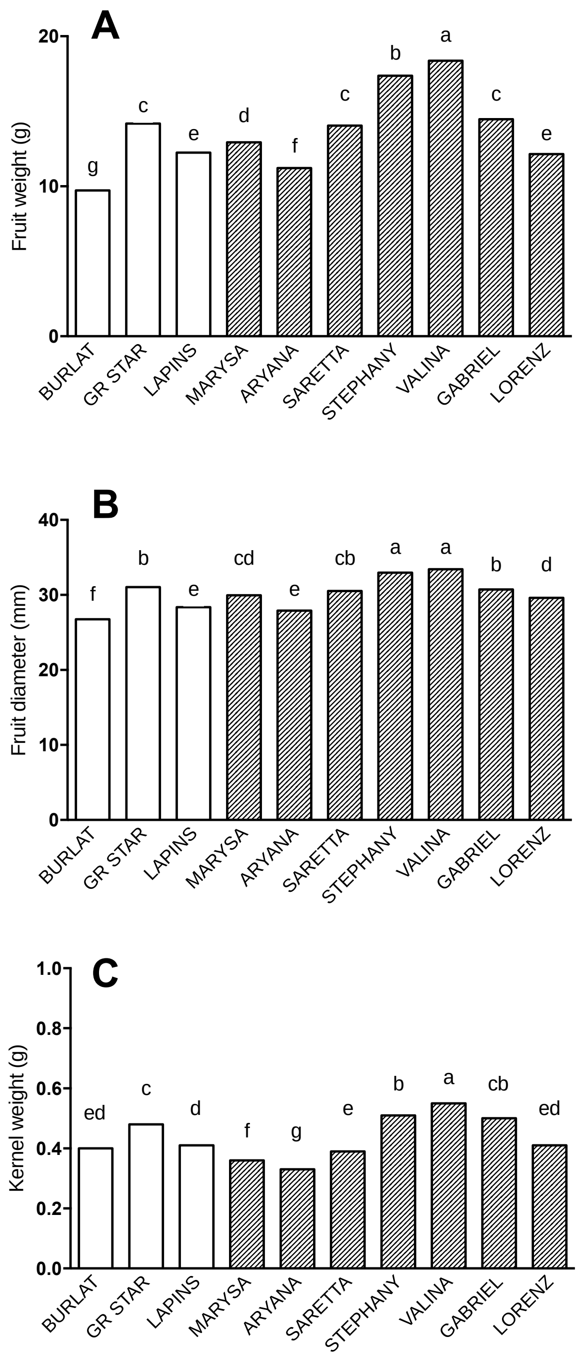

Figure S1. (**A**) Fruit weight; (**B**) fruit diameter; and (**C**) kernel weight of the different cultivars.

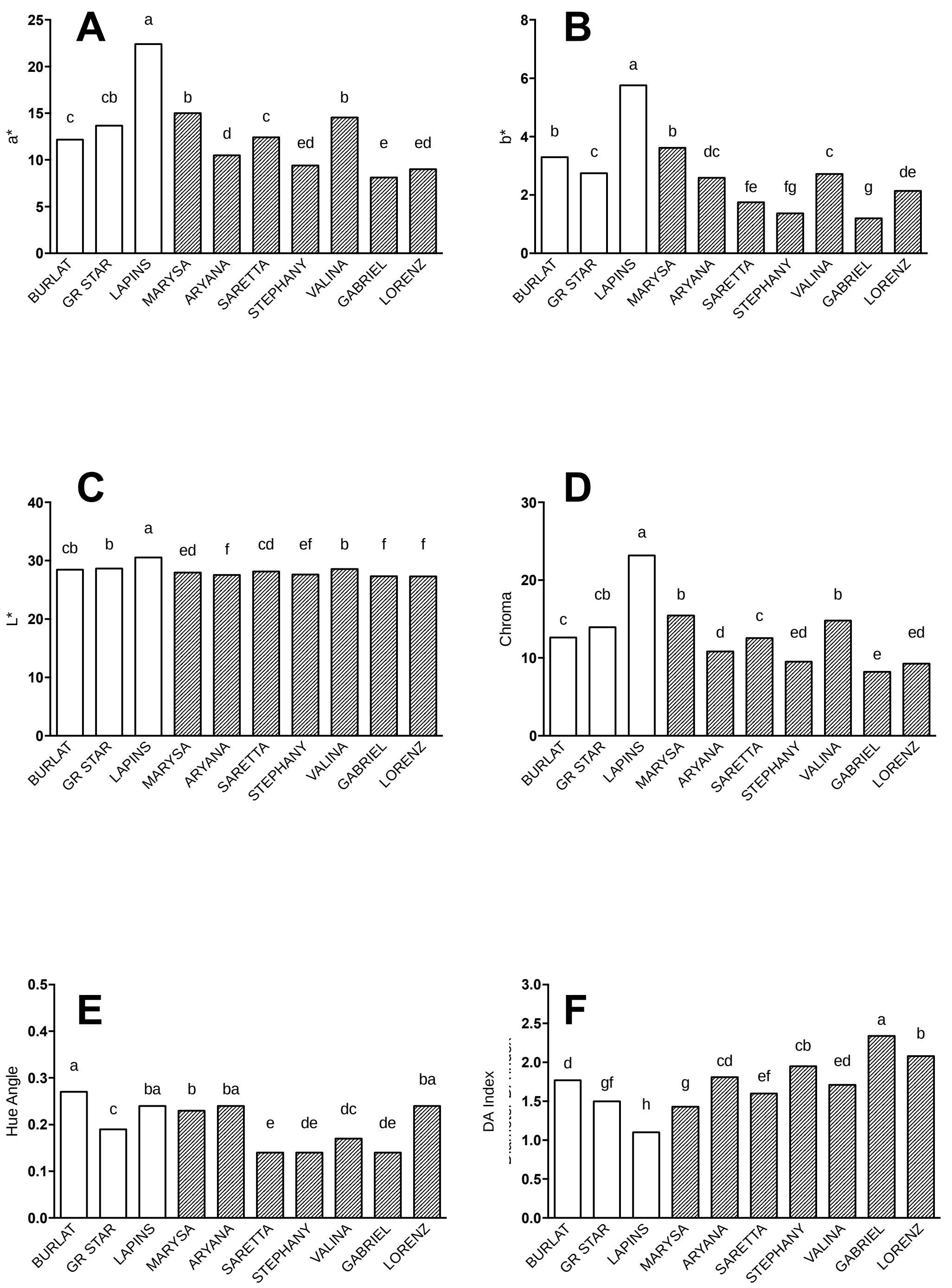

Figure S2. Color parameters of the different cultivars: (**A**) a*; (**B**) b*; (**C**) L*; (**D**) chroma; (**E**) hue angle; (**F**) DA index.

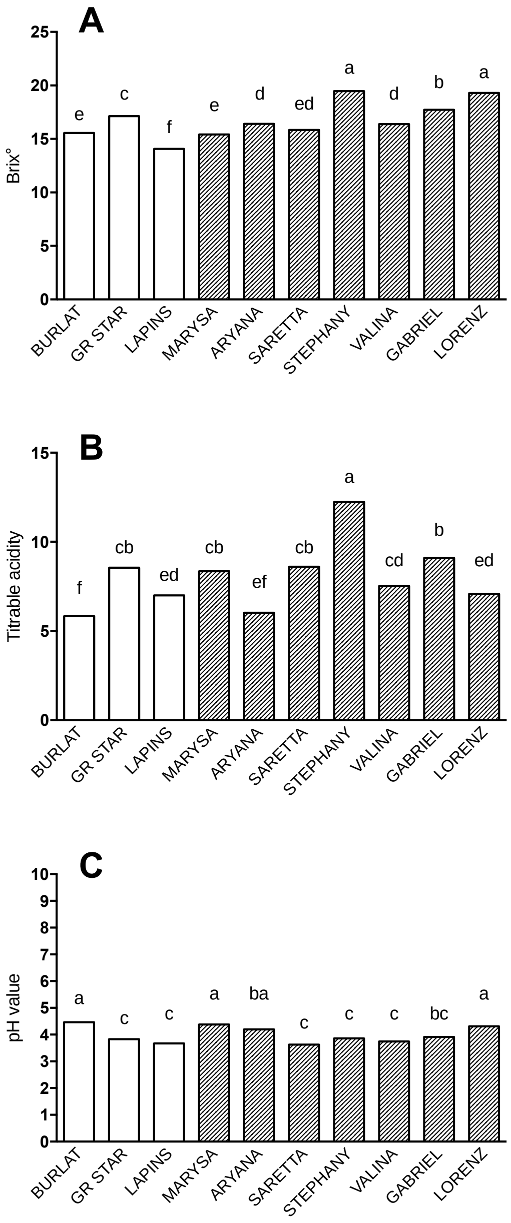

Figure S3. Other chemical-physical properties of the different cultivars: (**A**) total soluble solids; (**B**) titratable acidity; and (**C**) pH.

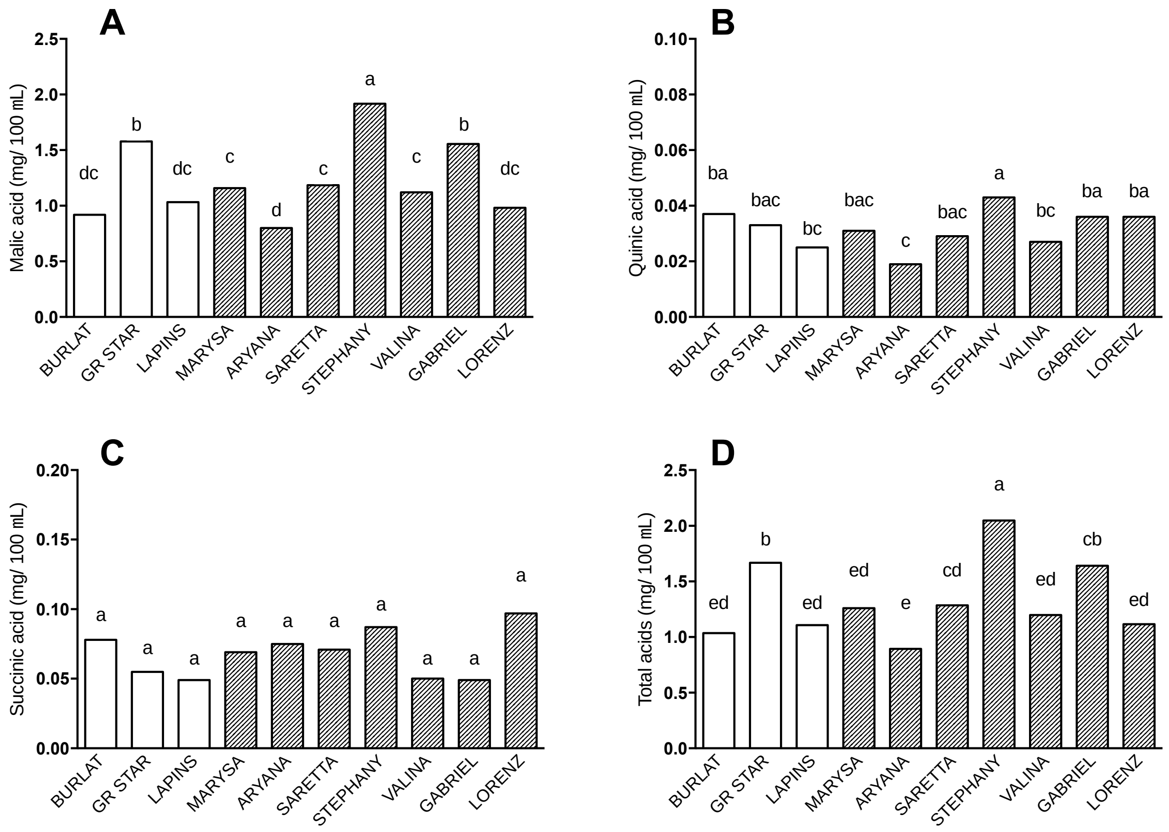

Figure S4. Results of the GC analysis of organic acids in the different cultivars: (**A**) malic acid; (**B**) quinic acid; (**C**) succinic acid; and (**D**) total acids by GC.

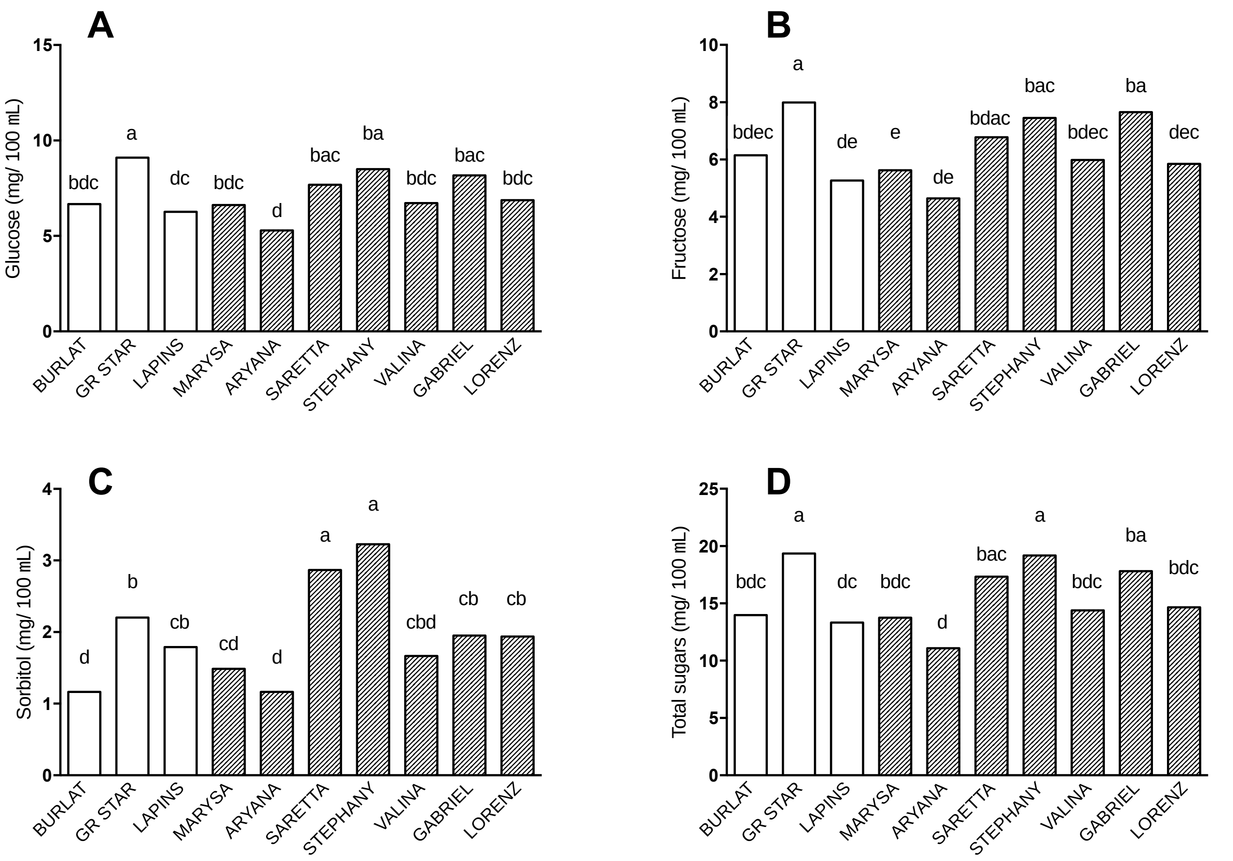

Figure S5. Results of the GC analysis of sugars in the different cultivars: (**A**) glucose; (**B**) fructose; (**C**) sorbitol; and (**D**) total sugars by GC.

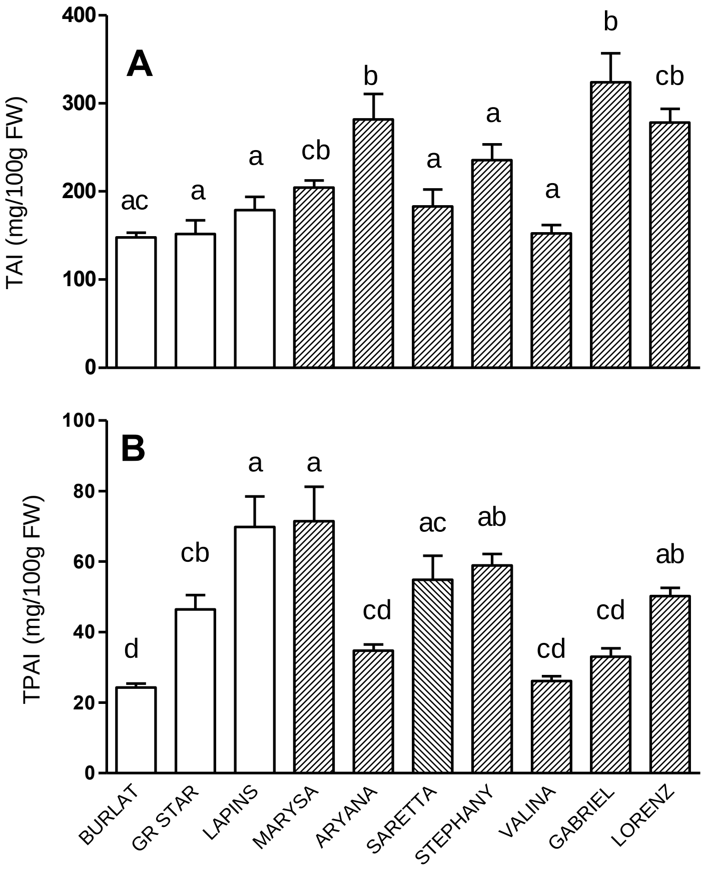

Figure S6. (**A**) Total Anthocyanin Index (TAI); and (**B**) Total Phenolic Acid Index (TPAI) in sweet cherry cultivars. Data are the mean ± SE of four biological replicates. Different letters indicate statistical significance ($p < 0.05$).

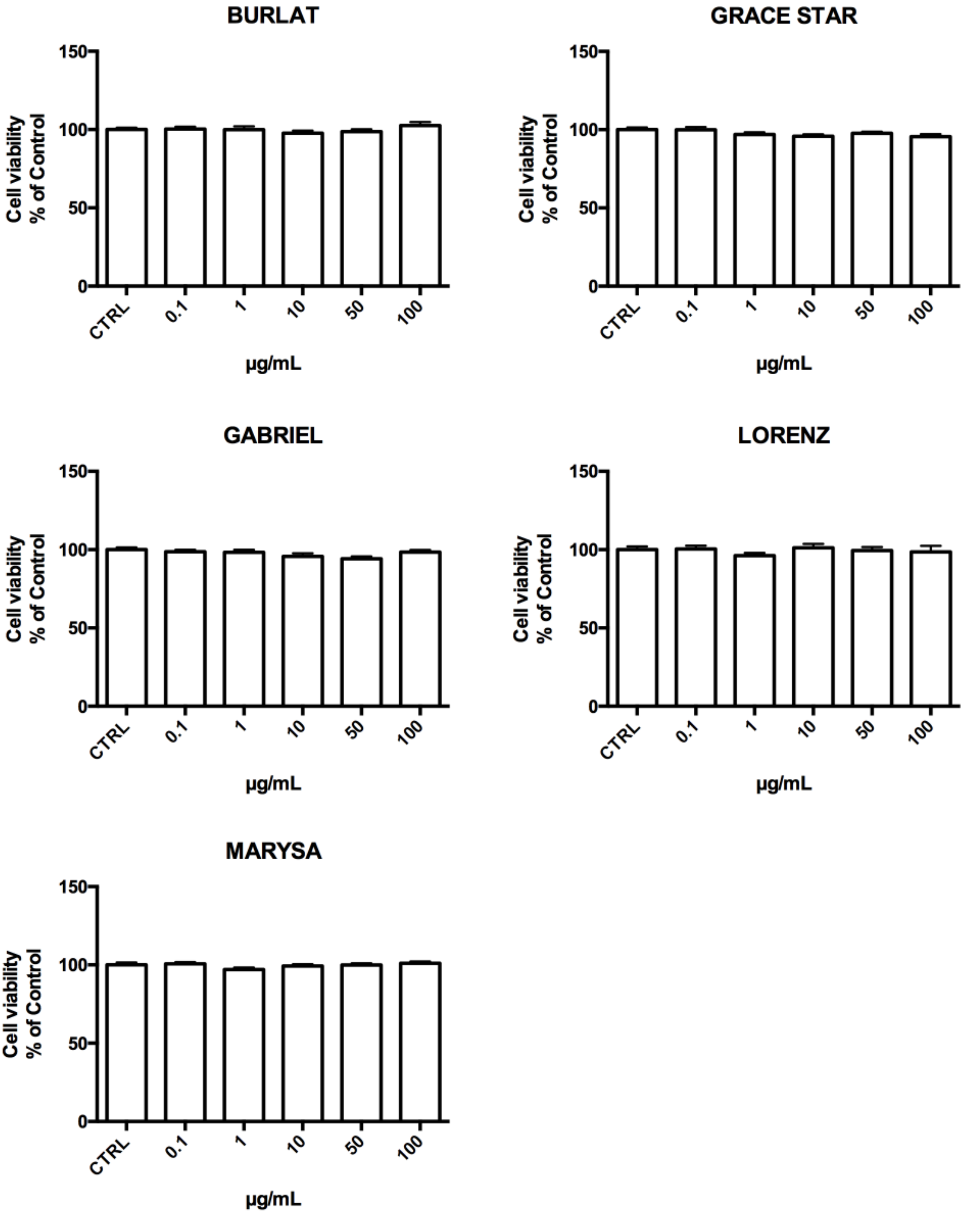

Figure S7. Effect of different concentrations of cherry extracts on SH-SY5Y cell viability as measured by the MTT assay. Each bar represents the mean ± SEM of at least three independent experiments. Data were analyzed by one-way ANOVA followed by Dunnett’s test.

Table S1. Correlations (Pearson's and p) among fruit quality traits in cherry cultivars.

Fruit trait	Fruit size	Fruit weight	Cherry DA Index	Durofel	Firmness	Soluble solids (Brix)	Fruit pH	Titratable acidity	L*	a*
Fruit size	1.0000 p= ---									
Fruit weight	0.9652 p=0.000	1.0000 p= ---								
Cherry DA Index	0.2122 p=0.189	0.1595 p=0.325	1.0000 p= ---							
Durofel	0.6744 p=0.000	0.6507 p=0.000	0.3117 p=0.050	1.0000 p= ---						
Firmness	0.1093 p=0.502	0.0147 p=0.928	0.1953 p=0.227	0.6207 p=0.000	1.0000 p= ---					
Soluble solids (Brix)	0.4811 p=0.002	0.3870 p=0.014	0.7396 p=0.000	0.5632 p=0.000	0.4487 p=0.004	1.0000 p= ---				
Fruit pH	-0.5112 p=0.001	-0.5839 p=0.000	0.2255 p=0.162	-0.4646 p=0.003	-0.0749 p=0.646	0.0712 p=0.662	1.0000 p= ---			
Fruit acidity	0.7007 p=0.000	0.6595 p=0.000	0.2389 p=0.138	0.5460 p=0.000	-0.0466 p=0.775	0.5273 p=0.000	-0.3779 p=0.016	1.0000 p= ---		
L*	-0.1946 p=0.229	-0.0884 p=0.588	-0.8111 p=0.000	-0.1441 p=0.375	-0.1626 p=0.316	-0.6909 p=0.000	-0.3937 p=0.012	-0.2527 p=0.116	1.0000 p= ---	
a*	-0.1897 p=0.241	-0.1057 p=0.516	-0.9022 p=0.000	-0.1605 p=0.322	-0.1240 p=0.446	-0.7655 p=0.000	-0.2964 p=0.063	-0.2736 p=0.088	0.9305 p=0.000	1.0000 p= [illegible]
b*	-0.4507 p=0.004	-0.3788 p=0.016	-0.8226 p=0.000	-0.2989 p=0.061	-0.0711 p=0.663	-0.7318 p=0.000	0.0095 p=0.954	-0.4756 p=0.002	0.8599 p=0.000	0.9227 p=0.0[illegible]
Chroma	-0.2076 p=0.199	-0.1239 p=0.446	-0.9012 p=0.000	-0.1706 p=0.293	-0.1223 p=0.452	-0.7671 p=0.000	-0.2789 p=0.081	-0.2871 p=0.072	0.9308 p=0.000	0.9997 p=0.0[illegible]
Hue angle	-0.7371 p=0.000	-0.7272 p=0.000	-0.3429 p=0.030	-0.5338 p=0.000	0.0320 p=0.844	-0.3644 p=0.021	0.6082 p=0.000	-0.7271 p=0.000	0.3191 p=0.045	0.3505 p=0.0[illegible]
Kernel weight	0.7764 p=0.000	0.7958 p=0.000	0.3168 p=0.046	0.6527 p=0.000	0.0534 p=0.743	0.4480 p=0.004	-0.4913 p=0.001	0.5312 p=0.000	-0.0221 p=0.892	-0.1408 p=0.3[illegible]

Table S2. Correlation (Pearson's and p) among fruit pH, fruit acidity, succinic acid, malic acid, quinic acid and total acid content in cherry cultivars.

Trait	Fruit pH	Fruit acidity	Succinic acid	Malic acid	Quinic acid	To[illegible]
Fruit pH	1.0000<br>p= ---					
Fruit acidity	-0.3779<br>p=0.016	1.0000<br>p= ---				
Succinic acid	0.2919<br>p=0.068	0.0358<br>p=0.826	1.0000<br>p= ---			
Malic acid	-0.3835<br>p=0.015	0.8643<br>p=0.000	0.1233<br>p=0.448	1.0000<br>p= ---		
Quinic acid	0.0817<br>p=0.616	0.4494<br>p=0.004	0.5321<br>p=0.000	0.6455<br>p=0.000	1.0000<br>p= ---	
Total acids by GC	-0.3510<br>p=0.026	0.8540<br>p=0.000	0.2020<br>p=0.211	0.9967<br>p=0.000	0.6886<br>p=0.000	[illegible]

Table S3. Correlation (Pearson's and p) among fructose, glucose, sorbitol, other sugars, total sugars, and soluble solids (Brix) in cherry cultivars.

Trait	Fructose	Glucose	Sorbitol	Other sugars	Total sugars	Soluble solids (Brix)
Fructose	1.0000<br>p= ---					
Glucose	0.9769<br>p=0.000	1.0000<br>p= ---				
Sorbitol	0.6804<br>p=0.000	0.7284<br>p=0.000	1.0000<br>p= ---			
Other sugars	0.5826<br>p=0.000	0.5576<br>p=0.000	0.2120<br>p=0.189	1.0000<br>p= ---		
Total sugars	0.9763<br>p=0.000	0.9877<br>p=0.000	0.8101<br>p=0.000	0.5268<br>p=0.000	1.0000<br>p= ---	
Soluble solids (Brix)	0.4106<br>p=0.008	0.4072<br>p=0.009	0.4642<br>p=0.003	-0.0325<br>p=0.842	0.4429<br>p=0.004	1.0000<br>p= ---

Table S4. Correlation (Pearson's and p) among anthocyanin levels, AA, and color parameters in cherry cultivars.

Trait	Cyanidin Glucoside	Cyanidin Rutinoside	Peonidin Glucoside	Peonidin Rutinoside	TAI	ORAC	L*	a*	b*	Chroma	Hue angle
Cyanidin Glucoside	1.0000 p= ---										
Cyanidin Rutinoside	0.0551 p=0.736	1.0000 p= ---									
Peonidin Glucoside	0.9390 p=0.000	0.0341 p=0.835	1.0000 p= ---								
Peonidin Rutinoside	-0.3839 p=0.014	0.5323 p=0.000	-0.3525 p=0.026	1.0000 p= ---							
TAI	0.3295 p=0.038	0.9595 p=00.00	0.2942 p=0.065	0.4336 p=0.005	1.0000 p= ---						
ORAC	0.5033 p=0.001	0.4603 p=0.003	0.5313 p=0.000	0.0382 p=0.815	0.5738 p=0.000	1.0000 p= ---					
L*	-0.1749 p=0.280	-0.5046 p=0.001	-0.1216 p=0.455	-0.3456 p=0.029	-0.5344 p=0.000	-0.1978 p=0.221	1.0000 p= ---				
a*	-0.2357 p=0.143	-0.4133 p=0.008	-0.1936 p=0.231	-0.3818 p=0.015	-0.4727 p=0.002	-0.1443 p=0.375	0.9305 p=0.000	1.0000 p= ---			
b*	0.0447 p=0.784	-0.2732 p=0.088	0.0725 p=0.657	-0.3857 p=0.014	-0.2603 p=0.105	0.0943 p=0.563	0.8599 p=0.000	0.9227 p=0.000	1.0000 p= ---		
Chroma	-0.2183 p=0.176	-0.4071 p=0.009	-0.1769 p=0.275	-0.3839 p=0.014	-0.4620 p=0.003	-0.1294 p=0.426	0.9308 p=0.000	0.9997 p=00.00	0.9318 p=0.000	1.0000 p= ---	
Hue angle	0.5989 p=0.000	0.0131 p=0.936	0.5957 p=0.000	-0.3054 p=0.055	0.1742 p=0.282	0.4627 p=0.003	0.3191 p=0.045	0.3505 p=0.027	0.6678 p=0.000	0.3719 p=0.018	1.0000 p= ---
